# Characterization of the complete chloroplast genome of *Xantolis weimingii* Huan C. Wang et Feng Yang et al. 2024 (Sapotaceae, Chrysophylloideae) and its phylogenetic implications

**DOI:** 10.1080/23802359.2026.2619330

**Published:** 2026-01-24

**Authors:** Dongrou Li, Shaoyun Liu, Jiangquan Wang, Feng Yang

**Affiliations:** ^a^ School of Environmental and Ecology, Hunan Agricultural University, Hunan, China; ^b^ School of Ecology and Environmental Science, Yunnan University, Kunming, China

**Keywords:** *Xantolis weimingii*, Chrysophylloideae, chloroplast genomics, phylogenetic analysis

## Abstract

*Xantolis weimingii* is a species recently described in 2024. In this study, we reported the complete chloroplast (cp) genome of *X. weimingii*, representing the first assembly of the genome of a *Xantolis* species. The chloroplast genome of *X. weimingii* was 158,458 bp in size, comprising a large single-copy (LSC) region of 87,778 bp, a small single-copy (SSC) region of 18,516 bp, and two inverted repeat (IR) regions of 26,082 bp each. A total of 114 distinct genes were annotated, including 75 of which are protein-coding genes, 30 tRNA genes, 4 rRNA genes, and five open reading frames. The Maximum Likelihood (ML) phylogenetic analysis showed that *X. weimingii* was sister to other members of the subfamily Chrysophylloideae with strong support.

## Introduction

*Xantolis* Raf. (Sapotaceae, Chrysophylloideae) comprises approximately 14 species, five of which occur in China, distributed from the eastern Himalayas to the Philippines in tropical Asia (van Royen 1957; Li 1987; Li and Pennington 2019). Morphologically, the genus is characterized by its obvious spines, lanceolate lobes of calyx and corolla, and aristate staminodes (Swenson and Anderberg 2005). Recent studies based on molecular data have demonstrated that the phylogenetic placement of *Xantolis* within the subfamily Chrysophylloideae remains unresolved, with conflicting topologies reported in previous analyses (Anderberg and Swenson 2003; Swenson and Anderberg 2005; Swenson et al. 2008, 2023; Bartish et al. 2011).

To date, only a handful of chloroplast genome sequences have been reported for Sapotaceae species (Liu et al. 2019; Niu et al. 2020; Zheng et al. 2020), and no complete chloroplast genome of any *Xantolis* species has been assembled. For poorly studied genera like *Xantolis*, chloroplast genome data can provide additional informative loci to improve the accuracy of phylogenetic inference. *Xantolis weimingii* Huan C. Wang et Feng Yang et al. (2024, Figure 1), a recently described species (Yang et al. 2024), is threatened by the ongoing clear-cutting and habitat destruction with only four populations discovered to date. In this study, we first reported the chloroplast genome of *X. weimingii*, which will offer a useful resource for future conservation genetics research.

**Figure 1.**
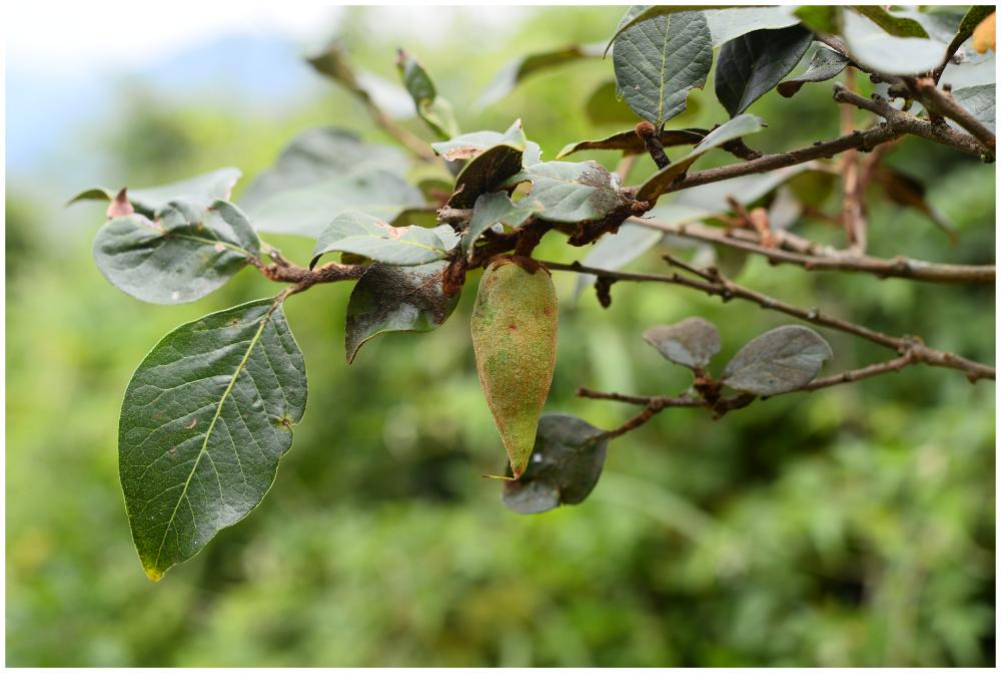
Picture of the collected sample of *Xantolis weimingii*. Photographed by Huanchong Wang on 17 September 2017. The young branches, leaves, petioles, pedicels, sepals and fruit of *X. weimingii* are covered with ferruginous arachnoid-lanate indumentum. The fruit has persistent sepals, a beak at the apex, and a persistent style.

## Materials and methods

Leaves of *X. weimingii* were collected from the living plants (Figure 1), from the mountain behind Fawu village, Dalongtan, Eshan County, Yuxi, Yunnan Province, China (sample collection site coordinates: 24°30’14.17ʺN, 102°03′46.60ʺE). The voucher specimen (contact: Feng Yang; yfde828@qq.com) was deposited at the Herbarium of Yunnan University (YUKU) under the voucher number ES2450.

Total genomic DNA was extracted from silica-dried leaves using a TIANGEN plant genomic DNA extraction kit (TIANGEN Biotech., Beijing, China) following the manufacturer’s protocols. Sequencing was performed on an Illumina HiSeq 6000 sequencing platform (Illumina, CA, USA) at Beijing Novogene Technology Co., Ltd. (Tianjin, China), generating 3 Gb of raw data for *X. weimingii*. The raw reads were processed in Trimmomatic v.0.32 (Bolger et al. 2014) to remove adapters, low-quality reads and reads containing unknown bases. The chloroplast genome of *X. weimingii* was assembled using GetOrganelle v1.7.7.1 (Jin et al. 2020). The assembled plastome sequence was annotated using the online annotation tools GeSeq (Tillich et al. 2017) and CPGAVAS2 (Shi et al. 2019), validated with CPGView (Liu et al. 2023), and adjusted with Geneious Prime 2020.0.3., ensuring annotation accuracy. Finally, the sequence was submitted to GenBank of NCBI. The circular chloroplast genome map of *X. weimingii* was also drawn using CPGview (Liu et al. 2023).

To clarify the phylogenetic position of *X. weimingii*, a total of 32 cpDNA related to *X. weimingii* from Sapotaceae, were used as ingroups. *Diospyros morrisiana* Hance was designated as the outgroup. All sequences were aligned using MAFFT v.7.490 (Katoh and Standley 2013) with the default parameters, and the aligned dataset was cleaned using TrimAl v.1.4.1 (Capella‐Gutierrez et al. 2009) to remove poorly aligned regions. Then we performed the maximum-likelihood (ML) phylogenetic analysis using IQ-TREE2 v. 2.1.3 (Nguyen et al. 2015) with branch support estimated using 1,000 replicates of the ultrafast bootstrapping algorithm (UFboot) (Minh et al. 2013), as well as the Shimodaira-Hasegawa-like approximate likelihood-ratio test (SHaLRT, Guindon et al. 2010). The phylogenetic trees were visualized in FigTree v.1.4.4 (Rambaut 2013).

## Results

**Figure 2.**
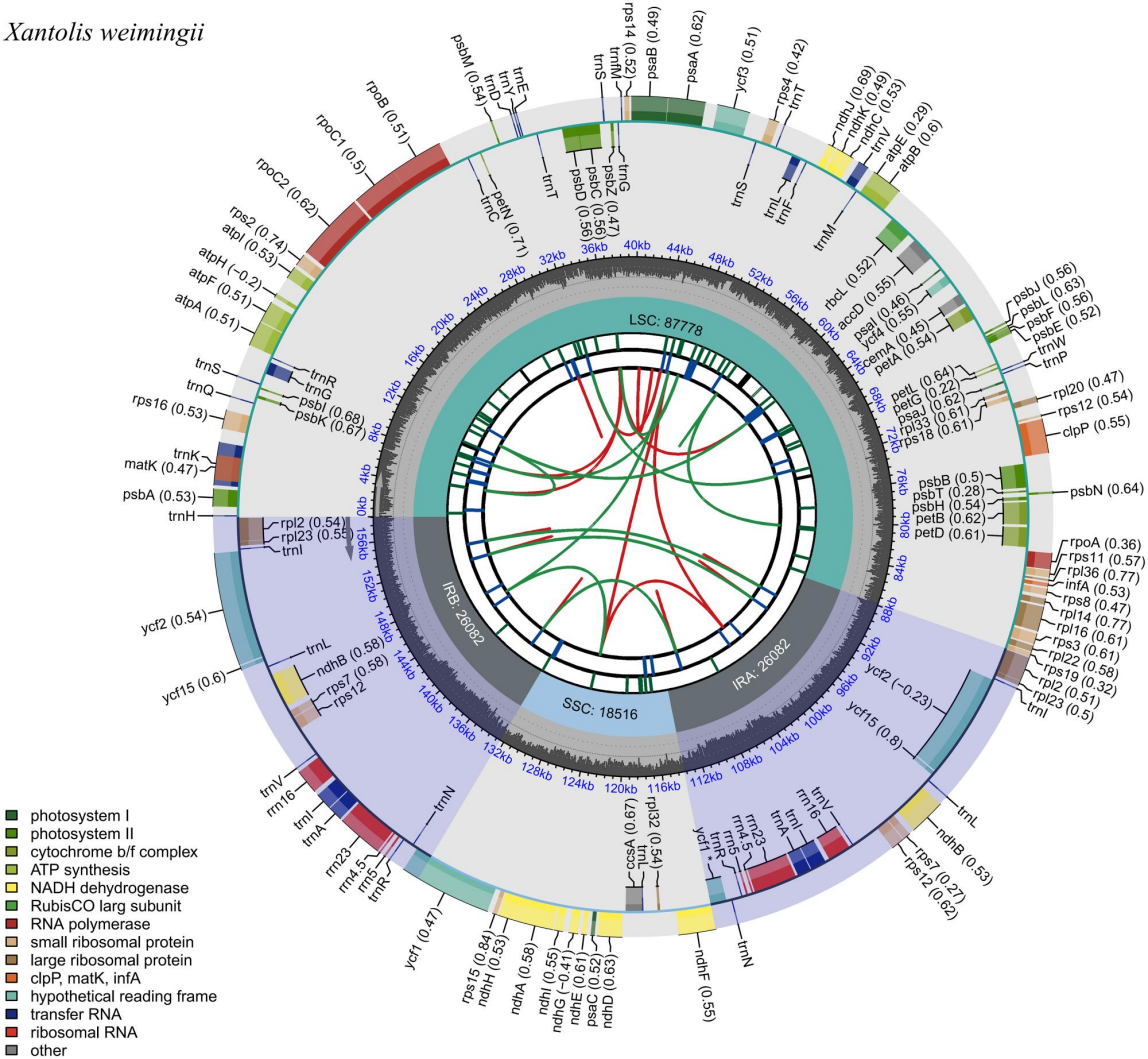
The chloroplast genome map of *Xantolis weimingii*. Genes inside and outside the circle are transcribed clockwise and counter-clockwise. The different colored boxes in the outermost circle show the genes. The inner circle has a grey area indicating the GC content, while the quadripartite structure (LSC, SSC, IRA, and IRB) is shown on the inner circle accordingly.

The chloroplast genome of *X. weimingii* was 158,458 bp in size with a 427.16× average depth of coverage (Supplemental Figure S1). It featured a circular and typical quadripartite structure, and contained a large single-copy (LSC, 87,778 bp) region, a small single-copy (SSC, 18,516 bp) region, and two inverted repeat (IR, 26,082 bp each) regions (Figure 2). The percentage of GC in the whole genome was 36.9%, and in LSC, SSC, and IR regions were  34.7%, 30.2%, and 42.9%, respectively. The chloroplast genome of *X. weimingii* had a total of 114 distinct genes, including 75 protein-coding genes (PCGs) and 30 tRNA genes, 4 rRNA genes and 5 open reading frames (ORFs) genes (Table 1). There were 15 genes, including 9 PCGs and 6 tRNA genes, with two exons and 3 genes (*ycf*3, *clp*P, and *rps*12) with three exons. Of the PCGs, 11 were cis-spliced genes, 9 of which contained one intron, and 2 contained two introns (Supplemental Figure S2). In addition, the location of the three exon regions of the trans-spliced gene *rps*12 were identified (Supplemental Figure S3).

**Table 1.** List of genes was annotated in the chloroplast genome of *Xantolis weimingii.*

Category	Gene group	Gene name	Unique genes number
Photosynthesis	Subunits of photosystem I	*psa* A, *psa* B, *psa*C, *psa*I, *psa*J	5
	Subunits of photosystem II	*psb*A, *psb*B, *psb*C, *psb*D, *psb*E, *psb*F, *psb*H, *psb*I, *psb*J, *psb*K, *psb*L, *psb*M, *psb*N, *psb*T, *psb*Z	15
	Subunits of NADH dehydrogenase	*ndh*A*, *ndh*B* (2), *ndh*C, *ndh*D, *ndh*E, *ndh*F, *ndh*G, *ndh*H, *ndh*I, *ndh*J, *ndh*K	11
	Subunits of cytochrome b/f complex	*pe*tA, *pet*B*, *pet*D*, *pet*G, *pet*L, *pet*N	6
	Subunits of ATP synthase	*atp*A, *atp*B, *atp*E, *atp*F*, *atp*H, *atp*I	6
	Large subunit of rubisco	*rbc*L	1
Self-replication	Proteins of large ribosomal subunit	*rpl*14, *rpl*16*, *rpl*2* (2), *rpl*20, *rpl*22, *rpl*23 (2), *rpl*32, *rpl*33, *rpl*36	9
	Proteins of small ribosomal subunit	*rps*11, *rps*12** (2), *rps*14, *rps*15, *rps*16*, *rps*18, *rps*19, *rps*2, *rps*3, *rps*4, *rps*7 (2), *rps*8	12
	Subunits of RNA polymerase	*rpo*A, *rpo*B, *rpo*C1*, *rpo*C2	4
	Ribosomal RNAs	*rrn*16 (2), *rrn*23 (2), *rrn*4.5 (2), *rrn*5 (2)	4
	Transfer RNAs	*trn*A-UGC* (2), *trn*C-GCA, *trn*D-GUC, *trn*E-UUC, *trn*F-GAA, *trn*G-GCC, *trn*G-UCC*, *trn*H-GUG, *trn*I-CAU (2), *trn*I-GAU* (2), *trn*K-UUU*, *trn*L-CAA (2), *trn*L-UAA*, *trn*L-UAG, *trn*M-CAU, *trn*fM-CAU, *trn*N-GUU (2), *trn*P-UGG, *trn*Q-UUG, *trn*R-ACG (2), trnR-UCU, trnS-GCU, *trn*S-GGA, *trn*S-UGA, *trn*T-GGU, *trn*T-UGU, *trn*V-GAC (2), *trn*V-UAC*, *trn*W-CCA, *trn*Y-GUA	30
Other genes	Maturase	*mat*K	1
	Protease	*clp*P**	1
	Envelope membrane protein	*cem*A	1
	Acetyl-CoA carboxylase	*acc*D	1
	c-type cytochrome synthesis gene	*ccs*A	1
	Translation initiation factor	*inf*A	1
Genes of unknown function	Conserved hypothetical chloroplast ORF	*ycf*1 (2)^#^, *ycf*2 (2), *ycf*3**, *ycf*4, *ycf*15 (2)	5

*Notes*. Gene *: Gene with one intron; Gene **: Gene with two introns; Gene #: One pseudogene identified; Gene (2): Number of copies of multi-copy genes.

**Figure 3.**
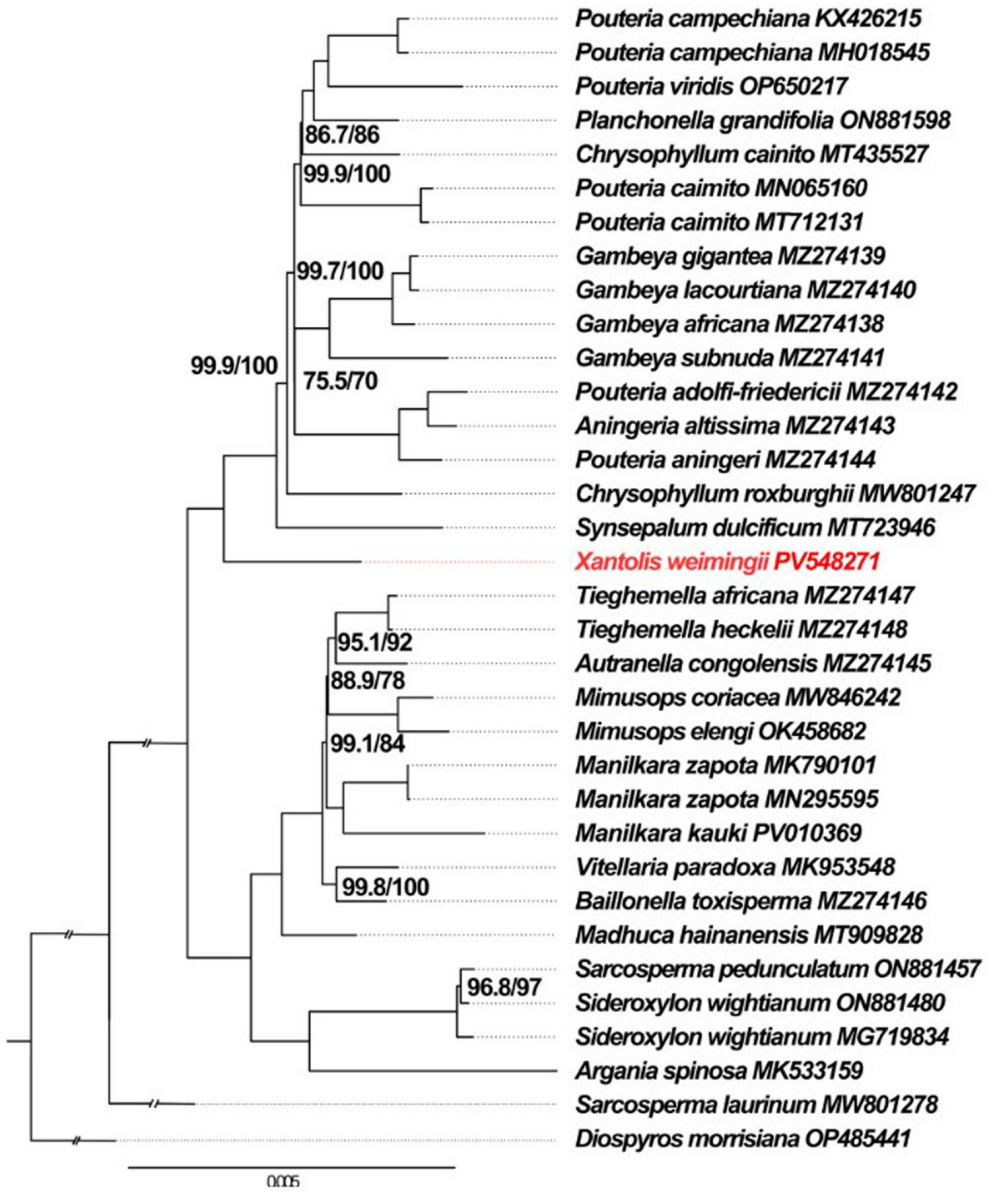
Molecular phylogenetic tree of 33 samples of Sapotaceae based on plastome sequences with maximum-likelihood analysis. Ultrafast bootstrap and Shimodaira-Hasegawa-like test values are marked near the nodes (UFboot/SH-aLRT). Nodes with 100 bootstrap percentages and 100 Shimodaira-Hasegawa-like test values are not marked on the tree. *Xantolis weimingii* is highlighted by using red colored text. *Diospyros morrisiana* (GenBank: OP485441) was used as outgroup. The following sequences were used: *Aningeria altissima* (GenBank: MZ274143, Mascarello et al. 2021), *Argania spinosa* (GenBank: MK533159), *Autranella congolensis* (GenBank: MZ274145, Mascarello et al. 2021), *Baillonella toxisperma* (GenBank: MZ274146, Mascarello et al. 2021), *Chrysophyllum cainito* (GenBank: MT435527, Zheng et al. 2020), *Chrysophyllum roxburghii* (GenBank: MW801247), *Gambeya africana* (GenBank: MZ274138, Mascarello et al. 2021), *Gambeya gigantea* (GenBank: MZ274139, Mascarello et al. 2021), *Gambeya lacourtiana* (GenBank: MZ274140, Mascarello et al. 2021), *Gambeya subnuda* (GenBank: MZ274141, Mascarello et al. 2021), *Madhuca hainanensis* (GenBank: MT909828), *Manilkara zapota* (GenBank: MK790101, MN295595, Li et al. 2019; Liu et al. 2019), *Manilkara kauki* (GenBank: PV010369), *Mimusops coriacea* (GenBank: MW846242), *Mimusops elengi* (GenBank: OK458682), *Planchonella grandifolia* (GenBank: ON881598), *Pouteria adolfi-friedericii* (GenBank: MZ274142, Mascarello et al. 2021), *Pouteria aningeri* (GenBank: MZ274144, Mascarello et al. 2021), *Pouteria caimito* (GenBank: MT712131, MN065160, Yang et al. 2019), *Pouteria campechiana* (GenBank: KX426215, Jo et al. 2016, MH018545, Niu et al. 2018), *Pouteria viridis* (GenBank: OP650217), *Sarcosperma laurinum* (GenBank: MW801278), *Sarcosperma pedunculatum* (GenBank: ON881457), *Sideroxylon wightianum* (GenBank: ON881480, MG719834), *Synsepalum dulcificum* (GenBank: MT723946, Niu et al. 2020), *Tieghemella africana* (GenBank: MZ274147, Mascarello et al. 2021), *Tieghemella heckelii* (GenBank: MZ274148, Mascarello et al. 2021), *Vitellaria paradoxa* (GenBank: MK953548, Wang et al. 2019), *Xantolis weimingii* (GenBank: PV548271, in this study).

The phylogenetic relationships of 33 samples were well resolved in this study using the chloroplast genome sequences (Figure 3). All 33 samples of Sapotaceae form a monophyletic clade with strong support (UFboot = 100%, SH-aLRT = 100%). The phylogenomic tree recovered three well-supported subfamily clades: the Sarcospermatoideae clade, the Sapotoideae clade, and the Chrysophylloideae clade. *Xantolis weimingii* occupied a basal position within Chrysophylloideae with strong support (UFboot = 100%, SH-aLRT = 100%).

## Discussion and conclusion

In this study, the chloroplast genome of *X. weimingii* was assembled and annotated, representing the first genomic resource not only for this rare species but also for the genus *Xantolis*. The chloroplast genome exhibits the typical quadripartite structure, with its length, GC content, gene composition and count showing highly similar to those of previously reported Sapotaceae species (Niu et al. 2020; Zheng et al. 2020), reflecting strong evolutionary conservation of plastid genomes in this family.

The phylogenetic results indicated that *X. weimingii* is the basal clade of Chrysophylloideae, and *Xantolis* represents an early-diverging lineage within the subfamily.  This finding can provide critical genomic evidence for resolving the ambiguous phylogenetic position of the genus. It was consistent with previous phylogenetic studies based on molecular markers (Anderberg and Swenson 2003; Swenson and Anderberg 2005; Swenson et al. 2008), but with greatly increased resolution. Overall, these results will provide effective molecular information for further studies on germplasm evaluation and molecular phylogeny of *Xantolis*.

## Supplementary Material

Figure S1. Clean reads coverage depth map of Xantolis weimingii.tif

## Data Availability

The genome sequence data supporting this study’s findings are openly available in GenBank of NCBI at https://www.ncbi.nlm.nih.gov/, reference number PV548271. The associated BioProject, SRA, and Bio-Sample numbers are PRJNA1254621, SRR33283946 and SAMN48116441, respectively.
