# Supplementary figures and images for: Characterization of the complete chloroplast genome of Xantolis weimingii Huan C. Wang et Feng Yang et al. 2024 (Sapotaceae, Chrysophylloideae) and its phylogenetic implications
Source: Mitochondrial DNA B Resour. 2026 Jan 24;11(2):290–4. doi: 10.1080/23802359.2026.2619330 (PMC12833893; doi:10.1080/23802359.2026.2619330)

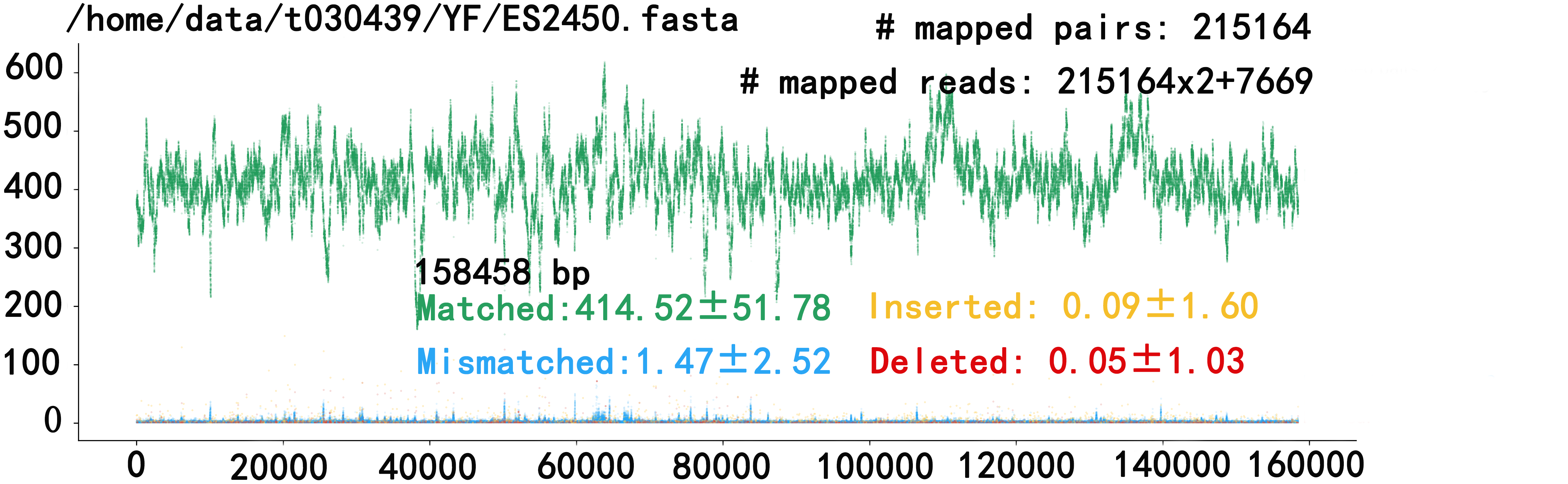

Supplement: Figure S1. Clean reads coverage depth map of Xantolis weimingii.tif [file TMDN_A_2619330_SM3546.tif]
